# Micro Energy Storage Systems in Energy Harvesting Applications: Analytical Evaluation towards Future Research Improvement

**DOI:** 10.3390/mi13040512

**Published:** 2022-03-25

**Authors:** Mahidur R. Sarker, Mohamad Hanif Md Saad, Amna Riaz, M. S. Hossain Lipu, José Luis Olazagoitia

**Affiliations:** 1 Institute of IR 4.0, Universiti Kebangsaan Malaysia, Bangi 43600, Selangor, Malaysia; hanifsaad@ukm.edu.my; 2 Industrial Engineering and Automotive, Nebrija University, Campus de la Dehesa de la Villa, Calle Pirineos, 55, 28040 Madrid, Spain; jolazago@nebrija.es; 3 Department of Electrical Engineering, Bahauddin Zakariya University, Multan 60000, Pakistan; amna.riaz@bzu.edu.pk; 4 Department of Electrical and Electronic Engineering, Green University of Bangladesh, Dhaka 1207, Bangladesh; shahadat@eee.green.edu.bd

**Keywords:** energy storage, energy harvesting, renewable energy, micro energy storage system

## Abstract

During the last decade, countless advancements have been made in the field of micro-energy storage systems (MESS) and ambient energy harvesting (EH) shows great potential for research and future improvement. A detailed historical overview with analysis, in the research area of MESS as a form of ambient EH, is presented in this study. The top-cited articles in the field of MESS ambient EH were selected from the Scopus database, and based on articles published from 2010 to 2021, and the number of citations. The search for these top-cited articles was conducted in the third week of December 2021. Mostly the manuscripts were technical and contained an experimental setup with algorithm development (65%), whereas 27.23% of the articles were survey-based. One important observation was that the top 20 selected articles, which are the most-cited articles in the different journals, come from numerous countries of origin. This study revealed that the MESS integrated renewable energy sources (RESs) are an enhancement field of research for EH applications. On the basis of this survey, we hope to identify and solve research problems in the field of MESS and RESs integration, and provide suggestions for future developments for EH applications.

## 1. Introduction

Nowadays, energy harvesting (EH) receives much attention due to the availability of abundant energy resources, the low cost of harvesters, and the reduction in the emission of greenhouse gases (GHG) [1,2]. In EH, either mega- or micro-scale, there are three important parameters that must be considered: a. the availability of the energy source (preferably free), b. the total cost of the harvesting system, and c. the impact of pollution on global warming. Due to EH fitting remarkably well within these parameters, it has gained in importanceto researchers globally [3]. Sarker et al. [3] provided a strategy to improve piezoelectric EH system efficiency in order to solve the problem of power loss. A major improvement in lowering the output error is made in terms of power efficiency, power loss, rising time, and settling time, to improve the proportional-integral voltage controller based on the hybrid optimization approach. Based on the findings, the overall efficiency of the piezoelectric EH system converter is estimated to be around 85%. However, it is difficult to design an EH system for specific applications due to the availability of different sources of energy, and the modeling of these systems for dealing with complex performance metrics [4,5]. Sarker et al. [4] discussed the effects of electromagnetic EH circuits, low-power electromagnetic EH devices, power electronic converters, and controllers in a variety of applications, as well as their merits and limitations. The authors also suggested a low-voltage, smart electronic circuit for a low-power sensor that gathers electromagnetic energy and that could deal with complex metrics. Riaz et al. [5] explained the importance of ambient energy as a reliable low-cost source for EH, and its vital role in the development of reliable and low-cost energy storage systems.

We present this recently published scientific literature in the area of MESS in terms of ambient EH, to highlight the latest trends and topics in these fields [6,7,8,9]. In the research field, the assessment and impact of published manuscripts is becoming popular. An impact factor is a measure of a journal’sor a researcher’s relevance to the literature and research in a specific field. In the research field, assessing the impact of a published manuscript is becoming popular; it’s related to the overview of the manuscript, keywords, and the author’s impact in the relevant field of research. In a specific field of research, the number of citations received is the measure of the impact of the manuscript, the authors, and publishers, and the influence of the research is measured using journal impact factors, citations to articles, and the h-index of researchers. According to a fundamental theorem, the impact factor is directly proportional to the number of citations. On the basis of this theorem, if we consider highly-cited articles in a specific field, then we can recognize the current exemplary work in that field, and would be able to predict the areas of future research in this field. In consideration of the most-cited articles, a few limitations can be investigated to help imagine new methods of design, modeling, and simulation. Older articles have more citations due to having been written some time ago, and this is also an important factor.

In the literature, many publications described MESS integrated RESs in EH application [10,11,12]. According to [10] the RES received attention because of their numerous advantages which include cost, reduced greenhouse effects, and the reliability of the system. When using batteries as MESS, voltage levels can be synthesized resulting inless harmonic distortion and noise in the output. However, the main disadvantage is that stronger semiconductor switches are required. The non-constant output capacitors, input current, and charging current, result in a high filter size, and extra electromagnetic interference concerns. Thermal energy is a renewable energy source and we are interested in energy on a micro-scale, such as the thermal energy of sun waves, vibrational energy, and the heat energy of the human body, among other resources [13,14,15].

Pusty et al. [15] discussed graphene, its derivatives in PVDF, and its co-polymers for efficient piezoelectric EH, as well as their production, characterization, characteristics, performance, and applications. PVDF and its copolymers are piezoelectric polymers that can be shaped into flexible EH devices. Also discussed by these authors was the electrospinning process for increasing the piezoelectricity of the graphene-PVDF nanocomposite. It is one of the most environmentally friendly energy sources available. Radiant energy is a type of energy that travels in the form of particles or waves; the most common examples are electromagnetic radiations, such as X-rays and heat released from a bonfire, microwave heat, gamma rays, and electricity [16]. In electromagnetic EH, the inductance determines the highest power transfer to the load, and this inductance can be utilized to tune resonance to obtain peak power. When nonlinearities are introduced into the EH system, however, it becomes difficult to obtain the maximum power. Radiant heat transmission is lethal and passers-by can be killed or injured by a fire’s electromagnetic radiation, which can also induce the combustion material to catch fire. The kinetic and potential energies, on the other hand, are the mechanical energy of an object. The energy produced by light waves, the energy produced by electricity, and the energy produced by sound waves are just a few examples of kinetic mechanical energy [17,18]. Cai et al. [17] determined that to charge batteries to higher levels, vibrations at high frequency are required. Mechanical energy’s biggest downside is its lack of safety and its difficulties in transmitting energy across great distances [19,20]. The power is generated using a variety of techniques and resources, both renewable and nonrenewable, but the major issue is the generation of low-cost electrical power, and the design of an energy storage device that avoids the drawbacks of ordinary batteries (self-discharging, loss of electrolyte, nickel gets stuck by hazards and burns faster, etc.). Only three options are available for storing the energy generated: batteries, fuel cells, and supercapacitors (SCs). SCs are now widely regarded as the most effective energy storage device. SCs outperform regular capacitors and secondary lithium-ion batteries [21]. In [22] the author suggests that a high level of energy self-sufficiency can be achieved utilizing currently available technologies. Microscale generation in the context of energy, is associated with high investment costs, but it has the potential to have a big ecological impact in the future.

In the most recent decade, the new trend of bibliographic analysis of the most-cited articles in different energy fields, has been reported, and some of these are; electromechanical EH system [22,23], thermal storage system with smart controlling strategies [24], electromagnetic storage system [25], piezoelectric EH system [26,27,28], application of hybrid materials in highly efficient energy storage systems (ESS) (e.g., SCs, batteries) [29,30,31,32], ESS optimization techniques [33,34], thermal ESS [35,36,37], electrical energy [38,39], and electrostatic ESS [40]. The Au NP–cellulose/polydimethylsiloxane nanocomposite was used to create a mechanical EH device such as a piezoelectric nanogenerator (PNG) invented by Pusty et al. [27]. When activated with a periodic force of 3 N, and a short circuit current of 700 nA, the PNG showed an open circuit voltage of 6 V. The advantage of the cellulose-based nanogenerator is that it allows for the production of a lead-free PNG. Because cellulose is biocompatible, biodegradable, and recyclable, it may enable the development of body-implanted EH devices that may capture mechanical energy from human body movements, blood circulation, and other sources. According to [32,34], a high level of energy self-sufficiency can be achieved utilizing currently available technologies. Micro-scale generation in the context of energy is associated with high investment costs, but it has the potential to have a big ecological impact in the future. The work done so far points in the right direction for selecting the right structure for a small, distributed energy system. The tremendous potential demonstrated in the case of integrating already known technologies, motivates future research, particularly in the field of management and control of tiny systems defined by a structure that grows in complexity with time. It is also a component of future studies that will be conducted. The current research [33] provides a new method for sizing a multi-source PV/Wind system with a hybrid energy storage system, and proposes an optimization technique that was developed by using detailed modeling of all sub-systems that make up the integral system. In addition, a frequency management method based on the Discrete Fourier Transform algorithm was utilized to disperse the power supplied by the power supply system into distinct dynamics. As a result, many frequency channels were collected in order to split the functions of each storage device, and demonstrate the impact of incorporating fast dynamics into renewable energy-based applications. In [39] the study proposed a better controller for micro EH systems.

Based on our analysis, this constitutes comprehensive research findings in the area of micro energy storage systems (MESS), from ambient EH systems, to power micro electronic devices [23,41,42]. In the last 20 years, improvement was based on the research and analysis in the field of MESS [36]. The aim of this study is to explain the importance of MESS integrated RESs, the application of EH, and to identify the issues and challenges in this specific field. For future developments, different suggestions are presented. To achieve these goals the methodology of this analysis involved the selection of the most highly-cited articles containing the important keywords. The document is reorganized based on the technique of using a sorting criterion known as, “time sighted–highest to lowest.” A filter was used on the Scopus data to locate the self-citations. The main goals of this study are illustrated below:

- To portray a clear picture of the research trends of the history and developments in the field of MESS and RESs.
- To preview a detailed analysis of the papers in the field of MESS as a form of RESs on the basis of number of citations.
- Highlight a few effective suggestions for future improvement in MESS.

## 2. Surveying Methodology

To highlight the importance of ESS in renewable EH, a brief analysis of the Scopus database was done in the third week of December 2021, considering the best research publications in the journal and indexed in the years 2010 to 2021. The main aim of this article is to provide a comprehensive study of the top-cited manuscripts covering future research trends in the field of MESS as a sort of RESs integration. In this analysis, highly-cited publication articles are selected and which contained a few keywords such as a. battery energy storage, b. RE and ambient EH. The English language filter was applied.

**Figure 1:**
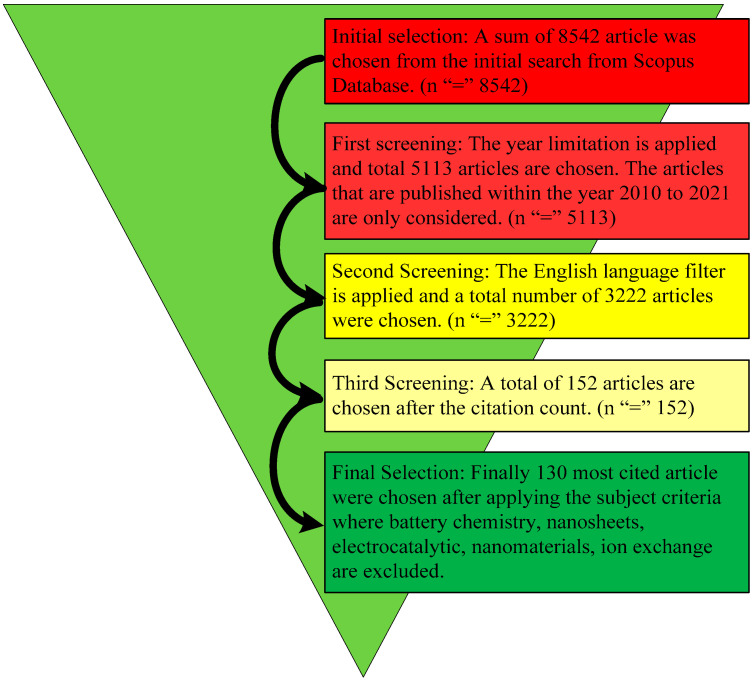
Schematic diagram of the reviewing method.

On the basis of the above strategy, and has already been stated, the manuscript was rearranged utilizing a sorting criterion known as, “time sighted–highest to lowest.” A filter was applied to the Scopus data to find the self-citations. Finally, the ultimate selection of appropriate articles was based on the titles, abstracts, and the contribution of the article to the field of MESS. This screening procedure is described in Figure 1.

### 2.1. Criteria to Analyze the Manuscript

After selecting the appropriate articles, the number of selected articles was reduced based on the top-cited manuscripts. Finally, we settled on 130 publications in the area of MESS as a form of RESs. The details of these articles are given below:

- Articles considering RE, battery ESS, and ambient EH.
- Exclusion was based on the topics of (i). battery chemistry, (ii). nanostructures, (iii). electrolytic analysis, (iv). nano materials, v. ion exchange, vi. composite analysis and (vii). nanowires.
- For the analysis, manuscripts published in the years 2010 to 2021 were included.
- Only manuscripts published in the English language were selected from the Scopus database.

### 2.2. Selection Process

In Figure 1, the selection process for ESS is explained in detail. After the initial selection, 8542 articles were chosen (n = 8542). The year range limitation was applied for the first evaluation and screening of the articles. On the basis of the year range limitation of between 2010 and 2021, a total of 3222 (n = 3222) articles were selected from the Scopus database.

### 2.3. Research Trends

Nowadays, there is a keenness among researchers to study the area of micro EH with renewable energy resources integration to create new resources for micro EH.

**Figure 2:**
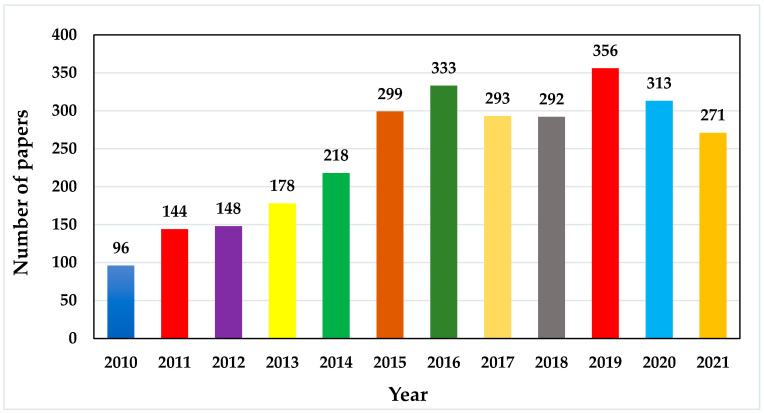
Trends in MESS research from the year 2010 to 2021.

As reported by the Scopus database, the first article on MESS as a sort of RESs integration, was published in 1978 [43]. After this publication, many researchers suggested and designed different techniques of harvesting with RESs for the MESS. Figure 2 presents the research trends in this field from 2010 to 2021. It is clear from Figure 2 that the trend of research in the field of MESS increased remarkably from 2015 to 2021; 2157 articles were published representing 73.34% of the total published articles. On the other hand, only 784 articles were published in the four years from 2010 to 2014, which is 26.66% of the total published articles.

### 2.4. Data Extraction

To carry out the analysis of research publications, data was extracted from the Scopus database based on the following parameters; (1) Title, (2) highest citation (last five years), (3) study types, (4) categories on the subject basis, (5) name of publisher, (6) total number of published articles in different journals, (7) impact factor, (8) country of origin, and (9) productive authors. After the extraction of articles based on these parameters, only 130 articles were chosen from the database.

### 2.5. Research Analyze and Outcomes

At the end of the initial search, 3222 articles were found. In the most-cited papers, the total citations were 23,499, ranging from 21 to 1928. Among these 130 papers, the citation of papers published in the last 5 years was 5512, and there were 12 articles having been cited more than 300 times. The most cited article was published in 2016 in the journal of “Chem. Soc. Rev.” with an impact factor of 54.564, and a total number of citations of 1928. After several screening processes, a total of 130 articles was selected, and Table 1 gives the complete information about the 130 articles including (i) author name, (ii) article DOI, (iii) impact factor, (iv) types of energy storage, (v) keywords, (vi) abbreviated journal name, (vii) name of publisher, (viii) year of publishing, (ix) country of origin, (x) number of citations.

## 3. Discussion

In the research of certain areas of technology, the first step is to gain knowledge and differentiate important information in the specific sector of study. The basic aim of this research is to present comprehensive information about recent study areas about MESS and RESs from the Scopus database, for future improvement. Future research directions in the PMEH include modeling, problem identification, simulation performance evolution, development, experimental prototype, an optimization method for sizing and increasing EH system efficiency, reviews, and technical overview. Table 1 provides complete information of the final 130 articles that were selected. Here, the top-cited manuscript was published in 2016 by Wang et al. [44], and the second most-cited article was published in 2011 by Sudevalayam et al. [45].

**Figure 3:**
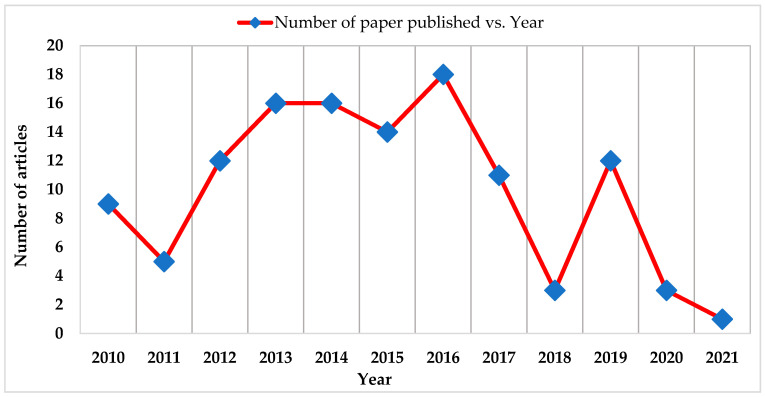
Distribution of 130 top cited manuscripts from 2010 to 2021.

In Figure 3 a graphical presentation of the total number of articles versus years is shown. It is clear from the graph that the articles published in the years 2017 and 2018 constituted only 9.17% and 2.5% of total publications, respectively. The arrangement of the final 20 highly-cited manuscripts per year in the area of MESS and ambient EH from 2010 to 2021, is presented in Figure 3. It is also clear that the lowest number of manuscripts (3) was published in 2020, followed by 2021 with 1 article as shown in Figure 3. Besides9 articles published in 2010, 16 articles were published in 2013 and 2014. Based on this analysis, we can suggest that the number of citations of a manuscript in some specific area of research is time-dependent.

The most-cited article is, “Electrochemical capacitors: mechanism, materials, systems, characterization and applications” by Wang et al. in the field of MESS and RE integration, published in the journal of “Chem. Soc. Rev.” in the year 2016, with a total number of citations of 1928 [44]. This paper reviews the latest research trends in SCs charge mechanism, electrode and electrolyte materials, SCs characterization methods and applications in ESS. The second most-cited article is also a survey review paper, “Energy Harvesting Sensor Nodes: Survey and Implications” by Sudevalayam et al. This paper reviews different aspects of EH sensor systems architecture, energy sources and different storage technologies [45]. This paper was published in 2011 in, “IEEE Communications Surveys and Tutorials” with a total number of citations at 1275. The third most-cited article was published in 2011, “Transmission with Energy Harvesting Nodes in Fading Wireless Channels: Optimal Policies” by Ozel et al. [46]. This article has 891 citations and was published in, “IEEE Journal on Selected Areas in Communications”. In this paper, the optimization of point-to-point data transmission with an EH transmitter is considered. The throughput of data is maximized, and the transmission time is reduced.

**Figure 4:**
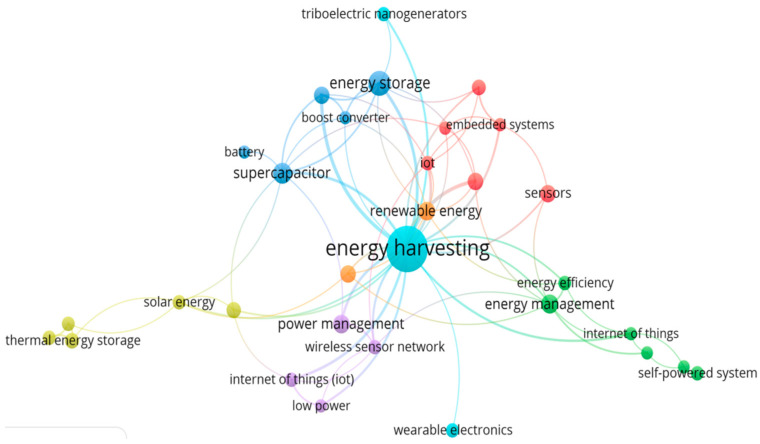
Finalized manuscript from the Scopus database based on the Co-occurrence keywords.

The analytical evaluation selection of the keywords from these manuscripts related to EH is shown in Figure 4 and Table 2, providing a clear view of the research contributions of these relatively new researchers in the said field. Figure 4 is developed with the help of VOSviewer (software) and represents a connection between all keywords. In this network the size of the label and circle measures the importance of the keyword, whereas the lines are used for connection between keywords to make a network. On the basis of this area of research and knowledge, various clusters are highlighted with the help of numerous colors. In this connective network it can be clearly observed that in the publications there is a strong relationship between (i) energy efficiency, (ii) energy management, (iii) self-powered system, and (iv) internet of things situated in the green cluster, whereas, in the blue cluster the (i) energy storage, (ii) boost converter, (iii) battery, (iv) supercapacitor are directly related to the energy harvesting. Renewable energy resources like solar and thermal energy are presented in the olive-green cluster which is also associated with SCs and power management. Ultimately, the red cluster represents the different types of systems discussed in MESS like; (i) embedded system, (ii) IoT, and (iii) sensors.

In Table 2, articles are selected based on 11 relevant, common keywords within the years 2010 to 2021. Based on the information presented in Table 2 the literature gaps and the research trend in the field of MESS as integration of RE could be highlighted. It is also clear from Table 2 that within the 11 relevant keywords, the highest common words are; “energy harvesting” followed by “renewable energy resources”, “hybrid EH and energy storage”, and “energy storage system”.

**Figure 5:**
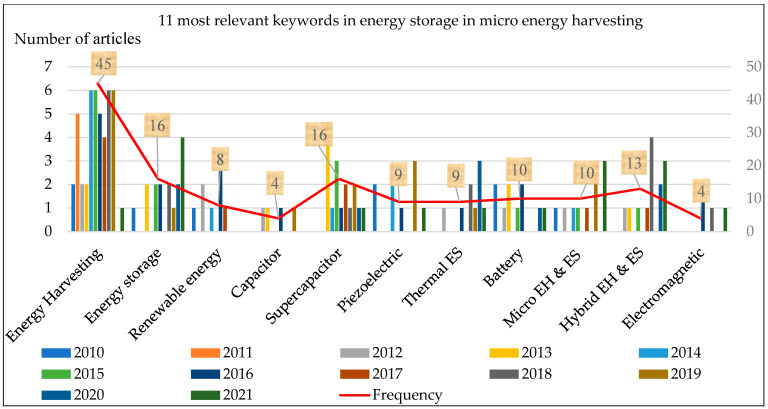
Presentation of 11 most relevant keywords from 2010 to 2021.

In the last 5 years, the most studied research keywords are, “energy harvesting”, “energy storage”, “supercapacitor”, “hybrid EH and energy storage”, and “batteries”, representing the current research trends in the area of ambient EH. The detailed distribution of the 11 relevant key research words is graphically represented in Figure 5 (graphical representation of Table 2. Here on the vertical axis, there are a number of articles and on the horizontal axis, the key research words are represented.

On the basis of Table 2 and Figure 5 it can be concluded that;

- Nowadays, hybrid energy storage related to RE e.g., vibrational energy, thermal, electromagnetic, etc., is well known.
- The popularity of EH (2014 to 2021), energy storage (2021), and hybrid EH and ES (2018 to 2021) has increased remarkably for the application of ESS and standalone devices.
- In MESS integrated RESs, SCs are the better option for energy storage compared to batteries.

The 10 manuscripts which are most-cited in the previous 5 years are listed in Table 3. The first most-cited article in Table 3 by Wang and the second manuscript by Sudevalayam.

Here, the number of citations per year for each manuscript is changing from the highest- to lowest-cited articles published by the researcher in the last 5 years. On the basis of Table 1, Table 2 and Table 3, it can be concluded that the manuscripts related to MESS in the field of ambient EH are the most-cited articles compared to the articles relating to capacitors and electromagnetic EH. Table 4 compares micro-energy storage systems such as batteries, capacitors, thermal storage, and ultra-capacitors.

The distribution of the most-cited manuscripts with frequency of publications, years range, and a number of citations are listed in Table 4. It can be observed from this table that the articles related to Review are highly-cited articles with 55.31% of citations in the Scopus database. The second most-cited articles are Modelling, Problem Identification, and Simulation Performance Evolution and have 23.99% of citations in the database. The third most-cited articles are related to Development and Experimental Prototype with citations 17.7%. In Table 5 there is a correlation between the frequency of publications and year range. It is clear from Table 5 that the articles published in the years 2010 to 2021 are mostly modeling, problem identification, and simulation performance evolution based, whereas the articles published in the years from 2013 to 2021 are mostly optimization methods for sizing and enhancing the efficiency of EH systems-based. Researchers are now concentrating their efforts on establishing a real-time application of MESS with RESs.

In Table 6 the distribution of highly-cited manuscripts in different categories of research is explained in detail. According to this table, the top-cited articles are based on “Micro energy storage system” and these are 34.84% of the total most-cited articles. The second most-cited articles are based on “Micro energy harvesting system” having 23.03% of the total most-cited articles. The articles on “Renewable energy source “account for 12.05%.

There is great potential for research in MESS integrated RE and battery storage systems. The recent research trends in 2021 focus on micro energy harvesting, hybrid EH and storage, and ESS.

According to the type of study (refer to Table 5), 40.52% of articles are based on modelling, problem identification and simulation performance evolution, development, and experimental prototype-based articles constitute 30.17%, whereas articles on optimization methods for sizing and enhancing the efficiency of EH system are 12.93%, review articles are 12.07% and articles on state-of-the-art technical overview are 4.31%.

**Figure 6:**
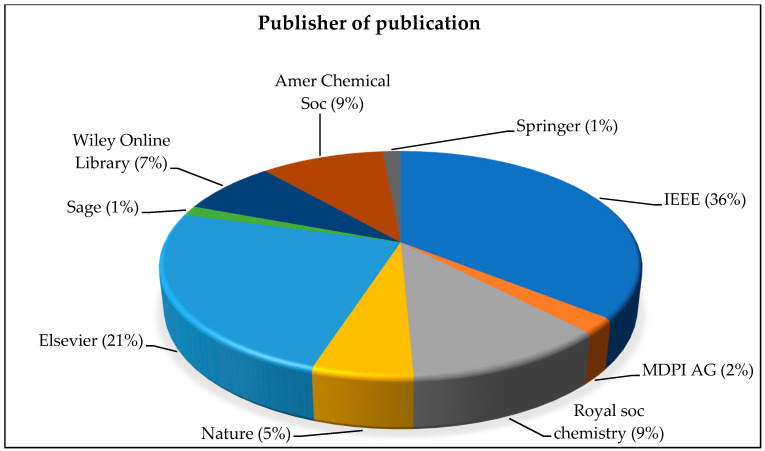
Frequency of different publisher articles.

Table 3, Table 5 and Table 6 concluded that in recent years the most focused article topics by researchers are: electrochemical capacitors; mechanism, materials, systems, characterization and applications, EH sensor nodes, and conformal piezoelectric EH and electrical energy storage. Figure 6 provides clear details of highly-cited manuscripts published by numerous publishers from the Scopus database.

The selected 130 articles are the most-cited and published in different peer-reviewed journals. A total of 34 different journals published these 130 most-cited articles with an impact factor from 2.34 to 46.495 (according to Journal Citation reports 2021). The journal “Applied Energy” published the 8 highest-cited articles, “Nano Energy” also published 8 articles, whereas “IEEE Transactions on Wired Communication” and “IEEE J. Solid State Circuits” published 7 articles each.

**Figure 7:**
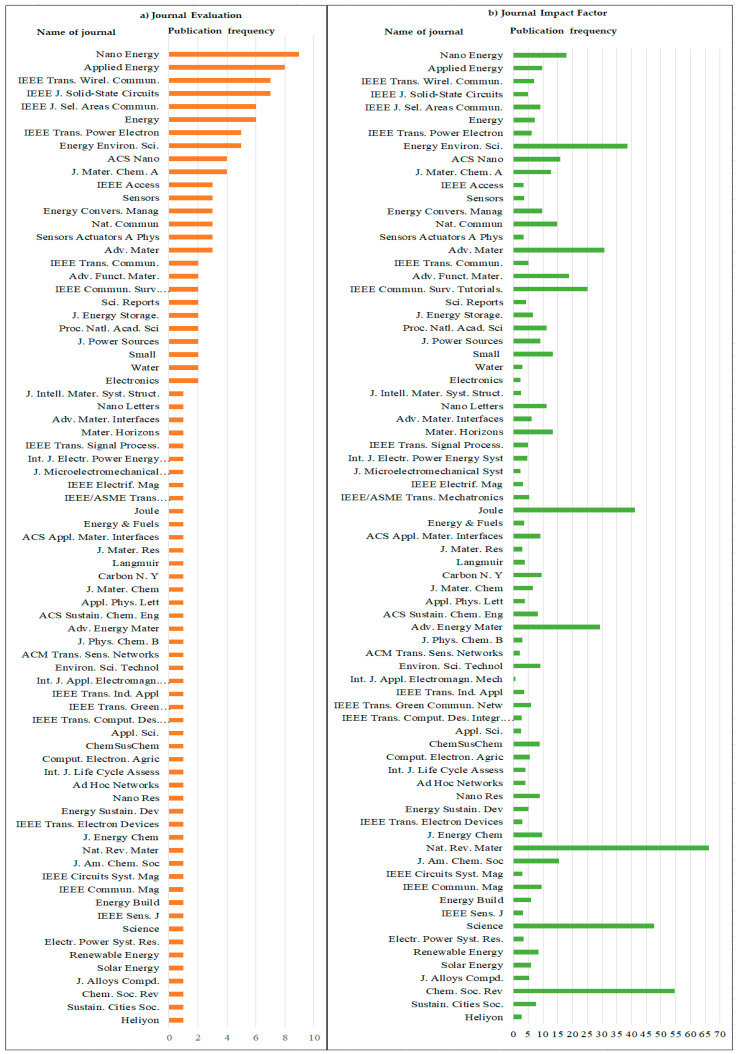
(**a**) Frequency of manuscripts published in different journals (**b**) Impact factor of this journal.

In the last 3 years “Elsevier” published the 290 highest-cited articles followed by “Royal Soc. Chemistry” with 4 articles. Both “Nano Energy” and “Energy Environ. Sci.” having 5 manuscripts from the highly-cited Scopus database. In Figure 7 below, the Evaluation of journals Figure 7a and Impact Factor Figure 7b, based on the frequency of publications, are described for the 130 selected publications. The highest impact factor is 38.53 but the frequency of publication is 4% for the journal of “Energy Environ. Sci.”, the second-highest impact factor is 30.85, with a frequency of publication of 3% for the journal of “Advanced Materials” whereas the article with the third-highest impact factor is “IEEE Communications Surveys and Tutorials” with an impact factor of 23.7 and 2% frequency of publications. On the basis of these details, we can conclude that the top 4 journals have the highest number of articles (40.23%) of the total selected most-cited articles with impact factors ranging from 5.55 to 15.13.

**Figure 8:**
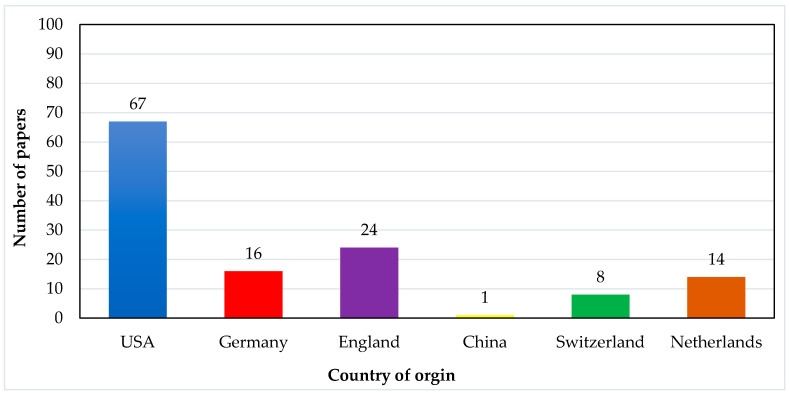
Graphical presentation of the number of papers vs. country of origin.

One of the interesting aspects is that of the selected 130 most-cited articles, 28 come from authors based in different countries (affiliation of the first author’s affiliation). The United States has the highest number of publications at 57.69%, England has the second-highest number of publications at 22.31%, and Germany has the third-highest number of publications at 8.46%. The remaining 11.54% of articles come from the rest of the world. In Figure 8 below, the distribution of most-cited articles based on country of origin is explained.

In Table 7, the highly-cited articles on the basis of most prolific authors, country of origin, number of articles, h-index, and the position on the author are listed. A. Yener from Ohio State University University, USA, is the author with the highest (4) number of most-cited publications. Yang H from Tsinghua University is the author with the second highest citations with 4 articles. Table 7 indicates that China has a second, third and fourth position in this list. A.Yener from the Ohio State University, USA, has the highest number of citations at 14,275 and a h-index of 55. Yang H from Tsinghua University has the second-highest number of citations (10,945) with a h-index of 48.

From the top-cited articles, 130 articles were selected, analyzed, and discussed, with the help of figures and tables. On the basis of this analysis and observations, we can conclude that in recent years the top-cited articles are “algorithm type” instead of “literature review”. The researchers are focusing on micro EH, micro-energy storage, renewable energy, and electrical storage systems. Among the selected total most- cited articles, only 12% of the articles are review based.

In this bibliometric analysis, we only considered the Scopus database and selected only 130 top-cited articles. For future work, the database of Web Sciences and Google Scholar should also be considered. To consider current research trends, research data from the years 2010–2021 was considered. In this analysis only articles written in the English language were considered; articles in other languages were ignored and this may have a global impact.

All the selected 130 articles have the same time in years for the citations. Finally, the papers that met the inclusion requirements were subjected to a subjective selection process. The combination of numerous disciplines makes the analysis difficult. The research-based battery analysis is included but the articles related to the batteries’ chemistry, and nano batteries are not considered. The citation analysis is the best way to understand current research trends in any specific field of research, limitations, and possible solutions. It is also a valuable way to distinguish the authors of the articles with high impact factors.

## 4. MESS Integrated with RESs: Issues and Challenges

The demand for an efficient, light and reliable MESS is growing as electronic gadgets, low-cost microelectronic devices, and WSNs become more common. Self-discharging, energy density, and longevity are all shortcomings of the current ESS. Although RESs are the best option as a source of energy, the insecurity of the energy supply is a challenge in harvesting energy from ambient sources. In many cases, due to the blast of Lithium-ion batteries, SCs are the best alternative of convection batteries for MESS.

### 4.1. Challenges Supercapacitor as MESS Integrated RESs

The energy density of SCs is low, resulting in a gap between SCs and batteries in terms of energy density. By increasing the electrode surface area of electrostatics double layer capacitors, the low energy density can be increased [177,178]. SCs have more discharging cycles than batteries. The reason for the low power is that the chemical reaction requires time to free electrons for current flow.

### 4.2. Thermal Issues of Batteries in MESS

The issues surrounding battery thermal management were discussed by various authors [179,180]. Chemical reactions generate high temperatures, which is a major issue that affects all batteries. A battery’s chemical properties are harmed by unusual temperatures, which results in considerable reductions in a battery’s efficiency. Secondary batteries require a temperature control system as well. The charging and discharging currents, as well as the battery’s power-handling capacities, are reduced by the impact of low temperature. The increasing temperature in the battery generates difficult circumstances that cause an abnormal chemical action and eventually lead to the battery exploding.

Although the stimulating effect can save some power, a higher current results in a higher temperature, which can lead to thermal runaway due to positive temperature feedback. Specific measures must be taken to prevent the battery from overheating. In comparison to other common batteries, the lithium-ion battery’s capacity increases as the temperature rises at the expense of the battery’s life. As a result, more attention is required to solve the battery’s thermal concerns in order to improve the efficiency of MESS integrated RESs.

### 4.3. Detection of Consistency

The SCs rated voltage is quite low (2.7 V), hence it is not suitable for large voltages [181]. The series combination is suggested for higher voltages. Overcharging also reduces the life of SCs. The performance of batteries is determined by two factors: the initial state of circuit (SOC) and temperature [182]. The constancy of lithium-ion batteries determines their performance. Partial discharge cycles can increase the performance of lithium batteries by reducing the battery temperature and avoiding discharging below 2 volts.

### 4.4. Environmental and Decarbonization Issues

Environmental factors like temperature and humidity have an impact on batteries and SCs [183]. Corrosion is a major issue for terminals. Carbon dioxide emissions have an impact on the environment. The US Environmental Protection Agency previously investigated lithium-ion batteries for their use of nickel and cobalt-based cathodes, as well as solvent-based electrode processing, and discovered significant environmental impacts, including resource depletion, global warming, ecological toxicity, and human health effects [184]. People who work with cobalt and nickel metal compounds in manufacturing or processing, or use them, may be at risk of respiratory, pulmonary, and neurological diseases. This risk can be reduced by recycling lithium-ion batteries to conserve natural resources and reduce the use of nickel and cobalt.

### 4.5. Industrial Standards

The use of SCs has increased due to its advantages of fast speed and short time requirement in development. Only some industries can design SCs to practical standards [185]. These industries also design and use batteries; strength, efficiency, and performance on standards. As a result, standards for (i) model name, (ii) terminology, (iii) electrical performance methodologies, (iv) general specifications, (v) safety technical criteria, and (vi) electrode material specifications and (vii) electrolyte specifications must be considered.

## 5. Conclusions and Future Improvement

Bibliometric analysis is a popular research method used for detecting the state of the art for MESS with RE integration. On the basis of this analysis, it was observed that bibliometric analysis is not only capable of utilizing quotative analysis but also capable of describing publication patterns within a given time period 2010 to 2021. The citation status of any research publication is the mirror image of citation, impact factor, and h-index. In this bibliographic review, 130 highly-cited articles covering the areas of MESS as a form of RESs with ambient EH were selected from the Scopus database. The selection criteria centred on the top-cited publications within the years 2010 to 2021. In this publication, different analyses have been presented and include: (a) distribution of most-cited articles in the last 5 years, (b) classification of publications on the basis of area of research, (c) study type, (d) country of origin and (e) journals publishing articles with highest citations. The basic idea behind this article is to understand and highlight the developments and problems in the areas of MESS in terms of ambient EH. In this bibliometric analysis, the thermal effect, cost minimization, and energy storage are also considered. On the basis of this study, it is analyzed that the MESS application is very useful in replacing traditional fuels with renewable energy resources. The MESS with ambient EH has disadvantages such as voltage variations, back feeding in the power system, system efficiency, future development of MESS, load, and power management. The top-cited articles also provide guidance for the proper solution of issues related to integration of RE with MESS connected to grid e.g., during the backup time the combo of battery-supercapacitor or battery hybrid system is the best solution of smoothing peak power during backup time. This research work also recommended that the use of MESS with ambient EH system will reduce the cost and emission of greenhouse gases (GHG), and it can also minimize the temperature effect, and ensure the dependability of micro EH application. Hence the chance of MESS as integration of RE of having a greater possibility to meet future demands. To solve the problem of thermal effect in electronics devices, a hybrid combination of battery with supercapacitor is the best option. The main target of MESS with ambient EH is to reduce the total cost of the system which also depends upon energy management. The MESS with RE integration use for micro electronic devices has proactive, systematic, and organized energy management. In the ESS technology, the future is low-cost carbon technology. MESS as integration of RE is the best alternative to fossil fuels which will reduce CO_2_ emission in the environment. For the reduction of carbon emission, lithium-ion batteries are a good choice. In this research article, the types of ESS with respect to cost, energies, power rating, output power, charging/discharging and environmental effect of recycling batteries and disposals. Here it is important to mention that the environmental effect of batteries could be reduced with the hybrid combination of battery and SCs. On the basis of this analysis, we can conclude that MESS integrated RES is the future of ambient EH. The attraction of MESS is low cost, easy to design, and has the availability of different optimization algorithms. Also, with these developments, the efficiency of the system can be increased remarkably.

This bibliometric analysis delivered numerous key and selective ideas for future research on the progress of MESS integrated RESs applications based on a careful review of existing research work. Here, the cost of ESS and Hybrid systems for MESS integrated with REs is discussed. To store the harvested energy, an efficient ESS is required and SCs are suggested as better storage devices than batteries due to the power density, cost in bulk and lifetime. The different problems in energy storage in MESS are discussed below and with the help of recent research studies, a few suggestions are presented to solve these problems.

- The SCs two fundamental problems are energy density and cost, which must be achieved without sacrificing exponential rate performance or high cycle life.
- The batteries must contend with cyclic life as well as rapid charging and discharging. A hybrid supercapacitor-battery system is thought to be a better solution for electrical energy storage. On the other hand, energy management is a challenge in a hybrid system. This issue can be solved with the help of an expert energy management system that extends battery life, improves system efficiency, and takes advantage of RESs.
- SCs are electronic devices that hold a small amount of energy. To solve this problem, a hybrid supercapacitor-lithium-ion battery system is excellent, since it not only increases the power capacity of MESS but also delivers a high-power density and energy density.
- During peak demand situations in electrical equipment, the batteries are put under a lot of strain. The hybrid ESS, which combines SCs and batteries, is the ideal solution.
- The manufacturing of SCs electrodes utilizing waste materials is the way of future improvement, but it will take a lot of research to get the optimum results.

This bibliometric analysis presents the following ideas, knowledge, and guidelines for future improvement and development in the areas of MESS integrated RES, EH, and different ESS:

- This study gives researchers a lot of information which helps them to publish research papers in well-known journals in the research area of MESS.
- For the relevant studies of MESS, RES, and EH, this document includes highly referenced papers and other possible articles. The characteristics of highly-cited papers can provide some insight into key advances in MESS. Citation reviews can be beneficial to the editorial board, potential writers, and reviewers by providing information on the types of publications that the researcher is interested in. It also provides writers with information on what makes a paper one of the most-cited.
- Keywords can be used to locate recent study papers from the past. It also indicates the breadth of relevant articles that have appeared in MESS integrated RES publications. The promotion of research keywords is expected to clarify the numerous phenomena in the field of MESS.
- Examination and interpretation of the manuscript submitted to the journal publishers and editors is made simple with this analysis.
- The rise of scientific cooperation, as well as the achievements of diverse authors, universities, and countries, has resulted in a massive mutual relationship. To get an article published, the writers and co-authors must make an original, descriptive, or empirical observation with those long-standing in the profession. In less developed countries, international collaboration fosters publications with higher citation counts. Furthermore, industrialized countries frequently reap the benefits of international cooperation.

In conclusion, based on the 130 most-frequently-cited publications over the last ten years, possible MESS studies and discoveries will not only play a critical role in the growth of evolving MESS technologies, but will also have significant implications on energy storage and harvesting market. We may be able to apply this analysis, knowledge, and information to overcome current ESS constraints and future developments in the field of MESS.

## Figures and Tables

**Table 1:** The 130 highly-cited manuscripts in the area of energy storage as a form of renewable energy.

Rank	Ref	Author Name	Article DOI	Impact Factor	Type of Energy Storage	Keywords	Abbreviated Name	Publisher	Publishing Year	Country	Number of Citation
1	[44]	Wang	10.1039/c5cs00580a	54.564	Capacitor energy storage	ES, EH	CSR	Royal soc chemistry	2016	England	1928
2	[45]	Sudevalayam	10.1109/SURV.2011.060710.00094	25.249	Battery energy storage	ES, EH	SURV	IEEE	2011	USA	1275
3	[46]	Ozel	10.1109/JSAC.2011.110921	9.144	Battery energy storage	ES, EH	JSAC	IEEE	2011	USA	891
4	[47]	Li	10.1002/adfm.201200591	18.808	Hybrid energy storage system	ES, EH	ADFM	WILEY	2012	Germany	676
5	[48]	Beaudin	10.1016/j.esd.2010.09.007	5.223	Electrical energy storage	ES, RES	ESD	Elsevier	2010	Netherlands	667
6	[49]	Bauer	10.1002/adma.201303349	30.849	Hybrid energy storage system	ESS, EH, RES	ADMA	WILEY	2014	Germany	574
7	[50]	Ho	10.1109/TSP.2012.2199984	4.931	Battery energy storage	ES, EH	TSP	IEEE	2012	USA	553
8	[51]	Dagdeviren	10.1073/pnas.1317233111	11.205	Hybrid energy storage system	ESS, EH, RES	PNAS	Natl Acad Sciences	2014	USA	527
9	[52]	Ramadass	10.1109/JSSC.2009.2034442	5.013	Capacitor energy storage	ESS, EH, RES	JSSC	IEEE	2010	USA	408
10	[53]	Ramadass	10.1109/JSSC.2010.2074090	-	Capacitor energy storage	ESS, EH, RES	JSSC	IEEE	2011	USA	393
11	[54]	El-Kady	10.1073/pnas.1420398112	11.205	Hybrid energy storage system	ESS, EH, RES	PNAS	Natl Acad Sciences	2015	USA	359
12	[55]	Lin	10.1021/nl4013002	11.189	Hybrid energy storage system	EH, RES	Nl	Amer Chemical Soc	2013	USA	331
13	[56]	Chang	10.1016/j.nanoen.2012.02.003	17.881	Hybrid energy storage system	EH, RES	NANOEN	Elsevier	2012	Netherlands	290
14	[57]	Akaydin	10.1177/1045389x10366317	2.569	Hybrid energy storage system	ES, AS	JIMSS	Sage	2010	England	284
15	[58]	Medepally	10.1109/TWC.2010.091510.100447	7.016	Hybrid energy storage system	EH, RES	TWC	IEEE	2010	USA	280
16	[59]	Zi	10.1038/ncomms10987	14.919	Hybrid energy storage system	EH, RES	NCOMMS	Nature	2016	Germany	245
17	[60]	Sun	10.1038/natrevmats.2017.23	66.308	Hybrid energy storage system	EH, ES	NATREVMATS	Nature	2017	Germany	241
18	[61]	Gunduz	10.1109/MCOM.2014.6710085	9.619	Hybrid energy storage system	EH, ES	MCOM	IEEE	2014	USA	238
19	[62]	Frackowiak	10.1016/S2095-4956(13)60028-5	9.676	Supercapacitors energy storage	EH, ES	EC	ELSEVIER	2013	Netherlands	218
20	[63]	Ng	10.1109/TWC.2013.052813.121589	7.016	Hybrid energy storage system	RES, EH	TWC	IEEE	2013	USA	195
21	[64]	Yang	10.1021/nn305247x	15.881	Hybrid energy storage system	RES, EH	NN	Amer Chemical Soc	2013	USA	193
22	[65]	Qian	10.1109/TPEL.2010.2043119	6.153	Hybrid energy storage system	RES, EH	TPEL	IEEE	2010	USA	190
23	[66]	Luo	10.1109/TWC.2013.012413.120488	7.016	Hybrid energy storage system	EH, ES	TWC	IEEE	2013	USA	182
24	[67]	Ozel	10.1109/TWC.2012.032812.110813	7.016	Battery energy storage	RES, EH	TWC	IEEE	2012	USA	175
25	[68]	Kim	10.1126/science.aam8771	47.728	Capacitor energy storage	EH, ES	SCIENCE	Amer Assoc advancement science	2017	USA	173
26	[69]	Ongaro	10.1109/TPEL.2012.2189022	6.153	Hybrid energy storage system	RES, EH, ES	TPEL	IEEE	2012	USA	170
27	[70]	Chen	10.1109/ISSCC.2010.5433921	-	Hybrid energy storage system	RES, ES	ISSCC	IEEE	2010	USA	158
28	[71]	Gorlatova	10.1109/TMC.2012.154	-	Hybrid energy storage system	EH, ES	TWC	IEEE	2013	USA	154
29	[72]	Pu	10.1002/smll.201702817	13.281	Battery energy storage	EH, ES	SMLL	WILEY	2018	Germany	150
30	[73]	Chai	10.1021/acsnano.6b05293	15.881	Supercapacitors energy storage	EH, ES	ACSNANO	Amer Chemical Soc	2016	USA	146
31	[74]	Soyata	10.1109/MCAS.2015.2510198	3.071	Hybrid energy storage	RES, EH	MCAS	IEEE	2016	USA	142
32	[75]	Dhillon	10.1109/TWC.2014.040214.131201	7.016	Battery energy storage	EH, ES	TWC	IEEE	2014	USA	142
33	[76]	Ramadoss	10.1021/acsnano.5b00759	15.881	Supercapacitors energy storage	EH, ES	ACSNANO	Amer Chemical Soc	2015	USA	141
34	[77]	Yu	10.3390/s140203323	3.576	Hybrid energy storage	EH, ES	Sensors	MDPI	2014	Switzerland	132
35	[78]	Andosca	10.1016/j.sna.2012.02.028	3.407	Battery energy storage	EH, ES, RES	SNA	Elsevier	2012	Switzerland	132
36	[79]	Jeong	10.1039/c4ee02435d	38.532	Hybrid energy storage	EH, RES	EES	Royal Soc Chemistry	2014	England	129
37	[80]	Adu-Manu	10.1145/3183338	2.253	Battery energy storage	EH, RES	TSN	Assoc Computing Machinery	2018	USA	128
38	[81]	Siddiqui	10.1016/j.nanoen.2015.04.030	17.881	Battery energy storage	EH, ES, RES	NANOEN	Elsevier	2015	Netherlands	128
39	[82]	Son	10.1039/c6ta03123d	12.732	Battery energy storage	EH, ES	MCA	Royal Soc Chemistry	2016	England	127
40	[83]	Aktakka	10.1109/JSSC.2014.2331953	5.013	Ultra-capacitor energy storage	EH, RES	JSSC	IEEE	2014	USA	122
41	[84]	Zhang	10.1016/j.apenergy.2016.06.054	9.746	Supercapacitors energy storage	EH, ES, RES	APENERGY	Elsevier	2016	England	121
42	[85]	Ostfeld	10.1038/srep26122	4.380	Battery energy storage	EH, ES, RES	SREP	Nature	2016	Germany	121
43	[86]	Niu	10.1016/j.nanoen.2014.05.018	17.881	Hybrid energy storage	EH, RES	NANOEN	Elsevier	2014	Netherlands	121
44	[87]	Lechêne	10.1016/j.nanoen.2016.06.017	17.881	Supercapacitors energy storage	EH, ES, RES	NANOEN	Elsevier	2016	Netherlands	171
45	[88]	Luo	10.1007/s12274-015-0894-8	8.897	Supercapacitors energy storage	EH, ES, RES	NR	Tsinghua Univ Press	2015	China	141
46	[89]	Moth-Poulsen	10.1039/c2ee22426g	38.532	Thermal energy storage	ES, RES	EE	Royal Soc Chemistry	2012	England	111
47	[90]	Chia	10.1109/TWC.2014.2339845	7.016	Hybrid energy storage	ES, RES	TWC	IEEE	2014	USA	108
48	[91]	Hehn	10.1109/JSSC.2012.2200530	5.013	Capacitor energy storage	EH, ES	JSSC	IEEE	2012	USA	107
49	[92]	Tan	10.1109/JSAC.2013.130715	9.144	Hybrid energy storage	EH, ES	JSAC	IEEE	2013	USA	106
50	[93]	Lv	10.1039/c8ee02792g	38.532	Hybrid energy storage	EH, ES	EE	Royal Soc Chemistry	2018	England	101
51	[94]	Yu	10.1021/jacs.5b03626	15.419	Battery energy storage	EH, ES, RES	JACS	Amer Chemical Soc	2015	USA	101
52	[95]	Dyatkin	10.1002/cssc.201300852	8.928	Supercapacitors energy storage	ES, RES	CSSC	WILEY	2013	Germany	101
53	[96]	Niu	10.1109/TED.2014.2377728	2.917	Capacitor energy storage	EH, ES, RES	TED	IEEE	2015	USA	100
54	[97]	Krikidis	10.1109/JSAC.2015.2479015	9.144	Battery energy storage	ES, RES	JSAC	IEEE	2015	USA	99
55	[98]	Yun	10.1016/j.nanoen.2019.03.074	17.881	Hybrid energy storage	EH, ES, RES	NANOEN	Elsevier	2019	Netherlands	98
56	[99]	Zhang	10.1039/c7ta00967d	12.732	Capacitor energy storage	EH, ES	JMCA	Royal Soc Chemistry	2017	England	98
57	[100]	Xia	10.1039/c6mh00159a	13.266	Battery energy storage	ES, RES	MH	Royal Soc Chemistry	2016	England	98
58	[101]	Pan	10.1109/INFCOM.2011.5934952	5.083	Hybrid energy storage	ES, EH	INFCOM	IEEE	2017	USA	95
59	[102]	Zhang	10.1016/j.enconman.2016.04.012	9.709	Supercapacitors energy storage	EH, ES, RES	ENCONMAN	Elsevier	2016	England	95
60	[103]	Fic	10.1039/c2jm35711a	6.626	Capacitor energy storage	ES, RES	JM	Royal Soc Chemistry	2012	England	94
61	[104]	Scalia	10.1016/j.jpowsour.2017.05.072	9.127	Supercapacitors energy storage	ES, RES	JPOWSOUR	Elsevier	2017	Netherlands	93
62	[105]	Sarı	10.1016/j.enbuild.2018.01.009	5.879	Thermal energy storage	ES, RES	ENBUILD	Elsevier	2018	Switzerland	91
63	[106]	Lei	10.1109/TGCN.2017.2684827	6.06	Battery energy storage	EH, RES	TGCN	IEEE	2017	USA	91
64	[107]	Wang	10.1021/nn4050408	15.881	Battery energy storage	ES, RES	NN	Amer Chemical Soc	2013	USA	91
65	[108]	Shigeta	10.1109/JSEN.2013.2264931	3.301	Capacitor energy storage	ES, EH	JSEN	IEEE	2013	USA	87
66	[109]	Ambaw	10.1016/j.compag.2012.05.009	5.565	Hybrid energy storage	ES, EH	COMPAG	Elsevier	2013	England	85
67	[110]	Zwerg	10.1109/ISSCC.2011.5746342	-	Battery energy storage	ES, EH	ISSCC	IEEE	2011	USA	84
68	[111]	Sakr	10.1109/JSAC.2015.2435358	9.144	Battery energy storage	EH, RES	JSAC	IEEE	2015	USA	83
69	[112]	Angrill	10.1007/s11367-011-0330-6	4.141	Hybrid energy storage	EH, RES	IJLCA	SPRINGER	2012	Germany	83
70	[113]	Prauzek	10.3390/s18082446	3.576	Hybrid energy storage	EH, ES, RES	S	MDPI	2018	Switzerland	82
71	[114]	Tutuncuoglu	10.1109/JSAC.2015.2391511	9.144	Battery energy storage	EH, ES	JSAC	IEEE	2015	USA	78
72	[115]	Prasad	10.1109/SURV.2013.062613.00235	25.249	Hybrid energy storage	EH, RES	SURV	IEEE	2014	USA	77
73	[116]	Anton	10.1177/1045389X14541501	2.569	Capacitor energy storage	EH, ES	JIMSS	SAGE	2014	England	76
74	[117]	Gasnier	10.1109/JSSC.2014.2325555	5.013	Capacitor energy storage	EH, ES	JSSC	IEEE	2014	USA	76
75	[118]	Hong	10.1002/adfm.201704353	18.808	Hybrid energy storage	EH, ES	ADFM	WILEY	2017	Germany	75
76	[119]	Farhat	10.1016/j.apenergy.2016.03.055	9.746	Hybrid energy storage	EH, RES	APENERGY	Elsevier	2017	England	75
77	[120]	Wang	10.1021/es300313d	9.028	Capacitor energy storage	EH, RES	EST	Amer Chemical Soc	2012	USA	74
78	[121]	Li	10.1016/j.nanoen.2019.03.061	17.881	Capacitor energy storage	ES, RES	NANOEN	Elsevier	2019	Netherlands	73
79	[122]	Song	10.1039/c6ta05816g	12.732	Supercapacitors energy storage	ES, RES	JMCA	Royal Soc Chemistry	2016	England	72
80	[123]	Chien	10.1002/smll.201403383	13.281	Supercapacitors energy storage	ES, RES	SMLL	WILEY	2015	Germany	72
81	[124]	Lakshminarayana	10.1109/JSAC.2014.2332093	9.144	Battery energy storage	ES, RES	JSAC	IEEE	2014	USA	72
82	[125]	Yang	10.1109/TPEL.2013.2238683	6.153	Supercapacitors energy storage	ES, RES	TPEL	IEEE	2013	USA	72
83	[126]	Samson	10.1016/j.sna.2010.12.020	3.407	Capacitor energy storage	ES, RES, EH	SNA	Elsevier	2011	Switzerland	72
84	[127]	Li	10.1038/srep02409	4.380	Hybrid energy storage	ES, RES	SREP	Nature	2013	Germany	71
85	[128]	Pampal	10.1016/j.jpowsour.2015.09.059	9.127	Battery energy storage	ES, RES	JPOWSOUR	Elsevier	2015	Netherlands	70
86	[129]	Song	10.1039/c5ta03349g	12.732	Supercapacitors energy storage	ES, RES, EH	JMCA	Royal Soc Chemistry	2015	England	70
87	[130]	Lallart	10.1063/1.3462304	3.791	Hybrid energy storage	ES, EH	APL	Amer Inst Physics	2010	USA	69
88	[131]	Liu	10.1109/TVLSI.2011.2159820	2.312	Ultra-capacitor energy storage	RES, EH	TVLSI	IEEE	2012	USA	68
89	[132]	Shen	10.1109/JMEMS.2017.2723018	2.417	Supercapacitors energy storage	ES, EH	JMEMS	IEEE	2017	USA	68
90	[133]	Amos	10.3390/w8040149	3.103	Hybrid energy storage	RES, EH	W	MDPI	2016	Switzerland	66
91	[134]	Wang	10.1109/TCAD.2015.2446937	2.807	Battery energy storage	ES, RES, EH	TCAD	IEEE	2016	USA	64
92	[135]	Michelusi	10.1109/TCOMM.2013.111113.130022	5.083	Battery energy storage	ES, EH	TCOMM	IEEE	2013	USA	64
93	[136]	Wickenheiser	10.1109/TMECH.2009.2027318	5.303	Capacitor energy storage	ES, EH	TMECH	IEEE	2010	USA	63
94	[137]	Li	10.1016/j.nanoen.2018.09.039	17.881	Capacitor energy storage	ES, EH	NANOEN	Elsevier	2018	Netherlands	62
95	[138]	El-Damak	10.1109/JSSC.2015.2503350	5.013	Battery energy storage	ES, EH	JSSC	IEEE	2016	USA	62
96	[139]	Zheng	10.1002/adma.201900583	30.849	Supercapacitors energy storage	ES, RES, EH	ADMA	WILEY	2019	Germany	61
97	[140]	Xiao	10.1016/j.joule.2019.09.005	41.248	Supercapacitors energy storage	RES, EH	JOULE	Cell Press	2019	USA	61
98	[141]	Allahbakhsh	10.1016/j.carbon.2019.04.009	9.594	Supercapacitors energy storage	ES, EH	CARBON	Elsevier	2019	England	60
99	[142]	Sherazi	10.1016/j.adhoc.2018.01.004	4.111	Hybrid energy storage	RES, EH	ADHOC	Elsevier	2018	Netherlands	60
100	[143]	Yu	10.1109/JSSC.2015.2476379	5.013	Capacitor energy storage	RES, EH	JSSC	IEEE	2015	USA	60
101	[144]	Tao	10.1039/c9ee00542k	38.532	Thermal energy storage	RES, ES	EE	Royal Soc Chemistry	2019	England	59
102	[145]	Pazhamalai	10.1002/admi.201800055	6.147	Supercapacitors energy storage	EH, ES	ADMI	WILEY	2018	USA	58
103	[146]	Liu	10.1557/jmr.2019.234	3.089	Supercapacitors energy storage	RES, ES	JMR	SPRINGER	2019	Germany	57
104	[147]	Wang	10.1016/j.apenergy.2018.08.080	9.746	Supercapacitors energy storage	RES, EH	APENERGY	Elsevier	2018	England	56
105	[148]	Abouzied	10.1109/JSSC.2016.2633985	5.013	Capacitor energy storage	RES, EH, ES	JSSC	IEEE	2017	USA	56
106	[149]	Kim	10.1109/TPEL.2012.2203147	6.153	Supercapacitors energy storage	RES, EH, ES	TPEL	IEEE	2013	USA	56
107	[150]	Agbossou	10.1016/j.sna.2010.06.027	3.407	Battery energy storage	RES, EH	SNA	Elsevier	2010	Switzerland	56
108	[151]	Lee	10.1109/TIA.2018.2799158	3.654	Battery energy storage	RES, EH, ES	TIA	IEEE	2018	USA	54
109	[152]	Tutuncuoglu	10.1109/ISIT.2013.6620495	-	Battery energy storage	EH, ES	ISIT	IEEE	2013	USA	54
110	[153]	Cansiz	10.1016/j.energy.2019.02.100	7.147	Hybrid energy storage	RES, EH	Energy	Elsevier	2019	England	54
111	[154]	Tempelaar	10.1021/jp510074q	2.991	Hybrid energy storage	EH, ES	JP	Amer Chemical Soc	2014	USA	53
112	[155]	Colin	10.1145/3173162.3173210	-	Hybrid energy storage	EH, ES	ACM	Assoc Computing Machinery	2018	USA	50
113	[156]	Dong	10.1016/j.nanoen.2017.10.035	17.881	Supercapacitors energy storage	EH, ES	NANOEN	Elsevier	2017	Netherlands	50
114	[157]	Yuan	10.1109/TWC.2014.2358215	7.016	Battery energy storage	EH, ES	TWC	IEEE	2015	USA	50
115	[158]	Lehtimäki	10.1016/j.ijepes.2014.01.004	4.630	Supercapacitors energy storage	EH, ES	IJEPES	Elsevier	2014	England	50
116	[159]	Mahidur	10.1016/j.sna.2019.111634	3.407	Hybrid energy storage	EH, ES	SNA	Elsevier	2019	Switzerland	50
117	[160]	Zhang	10.1038/s41467-020-16039-5	14.919	Battery energy storage	EH, ES	NCOMMS	Nature	2020	Germany	49
118	[161]	Mansø	10.1038/s41467-018-04230-8	14.919	Hybrid energy storage	RES, EH, ES	NCOMMS	Nature	2018	Germany	49
119	[162]	Yao	10.1021/acsami.6b07697	9.229	Capacitor energy storage	RES, EH, ES	ACSAMI	Amer Chemical Soc	2016	USA	48
120	[163]	Kimizuka	10.1021/acs.langmuir.6b03363	3.882	Solar Energy Storage	RES, EH, ES	LANGMUIR	Amer Chemical Soc	2016	USA	47
121	[164]	Chen	10.1002/aenm.201902769	29.368	Supercapacitors energy storage	RES, EH, ES	AENM	WILEY	2020	Germany	46
122	[165]	Shirvanimoghaddam	10.1109/ACCESS.2019.2928523	3.367	Hybrid energy storage	EH, ES	ACCESS	IEEE	2019	USA	46
123	[166]	Newell	10.1109/TPEL.2019.2894465	6.153	Hybrid energy storage	EH, ES	TPEL	IEEE	2019	USA	45
124	[167]	Jiang	10.1109/MELE.2014.2333561	3.217	Hybrid energy storage	RES, EH, ES	MELE	IEEE	2014	USA	44
125	[168]	Zhang	10.1016/j.apenergy.2015.11.096	9.746	Hybrid energy storage	RES, EH	APENERGY	Elsevier	2016	England	42
126	[169]	Tarelho	10.1016/j.mattod.2018.06.004	31.041	Supercapacitors energy storage	EH, ES	MATTOD	Elsevier	2018	England	36
127	[170]	He	10.1021/acssuschemeng.8b05606	8.198	Supercapacitors energy storage	EH, ES	ACSSUSCHEMENG	Amer Chemical Soc	2019	USA	32
128	[171]	Miao	10.1021/acs.energyfuels.1c00321	3.605	Supercapacitors energy storage	EH, ES	ENERGYFUELS	Amer Chemical Soc	2021	USA	26
129	[172]	Chen	10.1039/d0ee01355b	38.532	Thermal energy storage system	EH, ES	EE	Royal Soc Chemistry	2020	England	23
130	[173]	Mohamed	10.3233/JAE-150129	0.706	Hybrid energy storage system	EH	JAE	IOS Press	2016	Netherlands	21

**Table 2:** 11 most-selected keywords utilized in numerous manuscripts from 2010 to 2021.

Rank	Keywords	2010	2011	2012	2013	2014	2015	2016	2017	2018	2019	2020	2021	Frequency
1	Energy Harvesting	[57,58]	[45,46,71,110,126]	[50,130]	[66,92]	[61,75,86,115,116,167]	[81,96,111,123,143,157]	[74,82,134,138,162]	[60,99,106,118]	[113,137,142,147,151,169]	[98,108,140,141,153,166]	-	[5]	45
2	Energy storage	[48]	-	-	[127,152]	-	[97,114]	[59,85]	-	[38,155]	[39]	[35,174]	[6,22,25,40]	16
3	Renewable energy	[65]	-	[64,131]	-	[124]	-	[73,87]	[119]	-	-	-	-	8
4	Capacitor	-	-	[103]	[108]	-	-	[44]	-	-	[121]	-	-	4
5	Supercapacitor	-	-	-	[62,95,125,149]	[158]	[54,76,129]	[122]	[104,156]	[145]	[7,146]	[164]	[171]	16
6	Piezoelectric	[52,136]	-	-	-	[51,117]	-	[173]	-	-	[159,165,175]	-	[3]	9
7	Thermal energy storage	-	-	[89]	-	-	-	[163]		[105,161]	[144]	[24,36,172]	[37]	9
8	Battery	[53,70]	-	[67]	[107,135]	-	[94]	[100,138]	-	-	-	[160]	[8]	10
9	Micro EH and storage	[150]	-	[120]	-	[83]	[88]	-	[132]	-	[139,176]	-	[22,23,28]	10
10	Hybrid EH and energy storage	-	-	[69]	[63]	-	[54]	-	[101]	[29,30,33,93]	-	[9,32]	[8,31,34]	13
11	Electromagnetic	-	-	-	-	-	-	[102,168]	-	[170]	-	-	[4]	4

**Table 3:** Highly cited manuscripts in the last decayed.

Rank	DIO Number	Article Title	Last 5 Years Citation	Total Citation Rank	Ref No.	ACY	Advantage	Research Gap
1	10.1039/c5cs00580a	Electrochemical capacitors: mechanism, materials, systems, characterization and applications	1911	1	[44]	382	SCs have several orders of magnitude better energy storage capacity than normal dielectric capacitors. They have a high power density, long cyclic stability, and a high level of safety.	The energy storage capability of SCs is less than batteries.
2	10.1109/SURV.2011.060710.00094	Energy harvesting sensor nodes: Survey and implications	792	2	[45]	158	By utilizing recharge opportunities and adjusting performance settings based on current and expected energy levels, EH sensor nodes have the ability to solve the competing design goals of lifetime and performance.	Lifetime, cost, reliability, sensing, and transmission coverage are all difficult parameters to achieve in sensor networks using battery-powered nodes.
3	10.1109/JSAC.2011.110921	Transmission with Energy Harvesting Nodes in Fading Wireless Channels: Optimal Policies	366	7	[46]	72	Wireless systems with recharged nodes have a much longer lifespan and are more environmentally friendly. The ability of the nodes to capture energy during the duration of the transmission is a distinguishing feature of these systems.	The disadvantage is point-to-point optimization in data transmission in a wireless fading channel, which limits battery capacity.
4	10.1002/adfm.201200591	Hierarchically structured porous materials for energy conversion and storage	377	6	[174]	75	The established linkages between hierarchically porous structures and their energy conversion and storage performances can aid in the development of innovative structures with enhanced features.	The cost of hierarchically porous structures materials is high.
5	10.1016/j.esd.2010.09.007	Energy storage for mitigating the variability of renewable electricity sources: An updated review	400	4	[48]	80	Renewable resources cost is low.	Each challenge given by variable renewable resources necessitates a unique set of electrical energy storage features to handle the problem, and no single electrical energy storage technology consistently outperforms the others in varied applications.
6	10.1002/adma.201303349	25th anniversary article: A soft future: From robots and sensor skin to energy harvesters	446	3	[49]	93	EH is also favorable for Robotic applications.	Compex designing.
7	10.1109/TSP.2012.2199984	Optimal energy allocation for wireless communications with energy harvesting constraints	268	9	[50]	54	Considering channel conditions and uncertainty of RES the output can be maximized.	Renewable energy harvesting is an unreliable source of energy for sending data over a time-selective fading channel.
8	10.1073/pnas.1317233111	Conformal piezoelectric energy harvesting and storage from motions of the heart, lung, and diaphragm	399	5	[51]	80	Piezoelectric MESS may generate significant electrical power from the motions of inside organs, up to and above levels relevant for implant application.	Voltage output is also affected by the size of the heart, the velocity at which it beats, and the force with which it contracts.
9	10.1109/JSSC.2009.2034442	An efficient piezoelectric energy harvesting interface circuit using a bias-flip rectifier and shared inductor	228	10	[52]	46	Piezoelectric EH of ambient vibration energy is a prominent technique that can possibly deliver 10–100 s of µW of accessible power.	The interface circuitry of conventional piezoelectric harvesters is one of their major drawbacks.
10	10.1073/pnas.1420398112	Engineering three-dimensional hybrid SCs and microsupercapacitors for high-performance integrated energy storage	325	8	[54]	65	SCs overcome the limitation of energy densities.	It is necessary to develop a simple technique for fabricating supercapacitor arrays for high-voltage applications.

**Table 4:** A comparison of various micro-energy storage systems that are used in energy harvesting.

Different Micro Energy-Storage System	Objectives	Advantages	Disadvantages	References
Battery Storage System	Achieve high quality output voltages and input currents. Deliver a range of output volt-ages, ranging from much larger than the input voltage down to almost zero. Utilizing numerous sources and a single converter, create a multi-level voltage waveform using a split DC-link. To improve the efficiency and dependability of the system. A wide range of medium and high-voltage applications are available.	More voltage levels can be synthesised. Less harmonic distortion due to the stepped level voltage output. Less noise generation. Operation is simple at lower switching frequencies. Offers cost-effective solutions. External components that are smaller. Excellent waveform quality. As a result, filtering requirements are lowered. Superior capability for blocking voltage. Bidirectional switches can convert a constant ac input source to a variable voltage and variable frequency output.	More powerful semi-conductor switches are needed. The output capacitors, in put current and charging current are non-constant, resulting in a large filter size and additional electromagnetic interference concerns. The output is inverted, resulting in a more complicated sensing and feed-back circuit. Higher levels requires a larger number of diodes. The capacitor voltage cannot be maintained using the switching pattern chosen. Capacity is limited in or der to maximise output voltage. To decrease the high switching frequency harmonics, an input filter is required. Complicated, unreliable, and expensive.	[45,50,80,81,82]
Capacitor storage system	To improve the efficiency and dependability of the system. Allow DC motors to spin backwards or forwards. A high voltage step-up/step-down gain To keep their waveforms from intersecting as much as possible.	Lower output harmonic content. Power factor adjustment Control operation that is adaptable. Electromagnetic disturbances and voltage stress on semiconductor switches are reduced. Input current ripple is minimal. High productivity. Improved transient responsiveness Emissions of electromagnetic radiation are reduced. Easy to use controls.	Each cell requires a massive and expensive separate trans-former, as well as a voltage sensor. A large number of components requirements. Significant switching losses. Sensitive to changes in duty cycle. In the case of a high voltage need, a half-bridge converter is used. It has a higher voltage ripple than a half-bridge.	[44,52,53,68,99]
Thermal storage system	To achieve high efficiency and gain, use the shoot-through (ST) condition to raise the input voltage to higher levels. Optimizing several targets while minimising converter losses.	Proximity effect losses and lower winding costs. A wide variety of voltage gains. On the low-voltage side, low-current ripple Stresses caused by low voltage across power switches Reduced losses EMI is low Allows for high-volume operation. An extra clamping circuit is not necessary.	Inability to maintain higher efficacy over a wide range of output voltages. Complex control and structure. Its use should be limited. The ability to tolerate faults is limited. Gates with a high current rating. A large capacitor is necessary. Only operate in a boost or buck mode. The combined DC-DC boost converter and inverter system has a poorer dependability.	[89,105,144,172]
Super-capacitorstorage system	When compared to traditional converters, it delivers continuous in-put/out-put current.	Cost and volume reductions Low voltage load on the devices. High switching frequency excellent harmonic performance Lower switching losses, particularly when switching on the valley’s lowest point. Partially resonant with improved EMI. High efficiency and conversion ratio. It is inexpensive. Increased efficiency due to less transitional losses. Lower output voltage ripple Improved transient performance. Lower input capacitor ripple current rating requirements.	Virtual switch interface main switching devices are not interchangeable. As the load reduces, the frequency increases. Integrated trans-former with a lot of moving parts. High-priced controller. There are a lot of components required. Noise is a problem with switching converters. Analysis of complex systems under steady-state and transient conditions. Synchronization is difficult to achieve.	[62,73,76,84,87,88,95]

**Table 5:** Classification of a manuscript based on the type of research.

Research	Number of Publication	Years	Citation Range
Modelling, problem identification and simulation performance evolution	47	2010–2021	21–553
Development and experimental prototype	35	2010–2021	23–408
Optimization method for sizing and enhance efficiency of EH system	15	2013–2021	26–331
Review	14	2010–2021	60–1275
Technical overview	5	2012–2019	68–98

**Table 6:** Information about manuscripts in various areas of research.

Research Scope	References	Numbers	Citation Range
Micro energy harvesting system	[45,46,47,50,51,57,58,60,61,63,67,71,74,75,81,82,83,86,87,92,93,96,98,99,106,108,111,113,114,116,117,118,120,130,137,138,140,141,142,147,150,151,152,153,157,158,162,166,167,168,169,170]	52	32–1275
Micro energy storage system	[44,85,97,114,127,152,155]	6	50–1928
Piezoelectric energy harvesting system	[51,52,56,57,76,77,78,81,91,99,117,130,136,145,159,165,170,173]	18	21–527
Solar energy source	[64,73,87,94,100,104,119,121,144,156,161,163]	12	47–193
Thermal energy storage system	[44,89,105,161,172]	5	23–111
Electromagnetic energy harvesting	[102]	1	95
Battery energy storage	[67,69,70,107,135,138,160]	7	49–175
Renewable energy source	[48,65,84,90,124,131,147,168]	8	42–667
Photovoltaic Energy Harvesting	[69,123,134,143]	4	60–170
Thermoelectric energy-harvesting	[53,126,150]	3	56–393

**Table 7:** Top 10 authors with 2 or more published manuscripts.

Rank	Author’s Name	Institution	Country	Frequency of Manuscript	Citations	h-Index
1	Liu	Tsinghua University	China	13	5478	33
2	Wang	Fudan University	China	4	243	9
3	Hu	University of Pittsburgh	USA	6	2964	27
4	Yang, H	Tsinghua University	China	4	10,945	48
5	Xie, M	Tianjin University	China	5	859	14
6	A. Yener	Ohio State University	USA	4	14,275	55
7	Yu Li	Wuhan University of Technology	China	5	6323	38
8	Zareipour	University of Calgary	Canada	5	6821	41
9	Ho	Institute for Infocomm Research	Singapore	2	5302	22
10	Dagdeviren	Massachusetts Institute of Technology	USA	2	4352	21

## Data Availability

Not applicable.
